# A modified agar pad method for mycobacterial live-cell imaging

**DOI:** 10.1186/1756-0500-4-73

**Published:** 2011-03-21

**Authors:** Graham Joyce, Brian D Robertson, Kerstin J Williams

**Affiliations:** 1 Centre for Molecular Microbiology and Infection, Imperial College, London, SW7 2AZ, UK

## Abstract

**Background:**

Two general approaches to prokaryotic live-cell imaging have been employed to date, growing bacteria on thin agar pads or growing bacteria in micro-channels. The methods using agar pads 'sandwich' the cells between the agar pad on the bottom and a glass cover slip on top, before sealing the cover slip. The advantages of this technique are that it is simple and relatively inexpensive to set up. However, once the cover slip is sealed, the environmental conditions cannot be manipulated. Furthermore, desiccation of the agar pad, and the growth of cells in a sealed environment where the oxygen concentration will be in gradual decline, may not permit longer term studies such as those required for the slower growing mycobacteria.

**Findings:**

We report here a modified agar pad method where the cells are sandwiched between a cover slip on the bottom and an agar pad on top of the cover slip (rather than the reverse) and the cells viewed from below using an inverted microscope. This critical modification overcomes some of the current limitations with agar pad methods and was used to produce time-lapse images and movies of cell growth for *Mycobacterium smegmatis *and *Mycobacterium bovis *BCG.

**Conclusions:**

This method offers improvement on the current agar pad methods in that long term live cell imaging studies can be performed and modification of the media during the experiment is permitted.

## Background

Modern microscopy instrumentation and techniques permit the study of microorganisms at the single cell level and have been used extensively to study essential bacterial processes such as motility, cell division and the cell cycle. Several methods have been reported for live-cell time-lapse imaging of bacteria which can be crudely divided into two general approaches: growing bacteria on thin agar pads [1-3] or growing bacteria in micro-fluidic chambers [4,5]. Both have their advantages and limitations.

The use of micro-channels is a sophisticated technique, often combined with micro-fluidic devices, and is consequently a more expensive method that requires specialised equipment and greater operator expertise. A cover slip containing a micro-channel is produced and the cells/culture fluid added to the channel, which is then covered with a porous membrane [5]. In this technique, the system is not sealed so gaseous exchange can occur freely and agents can be added to the culture fluid mid-experiment, so longer and more varied manipulations can be performed. However, micro-fluidic devices usually constrain cell growth to one or two dimensions to allow for single cell tracking, and the cells have to be attached to a substratum strong enough to resist the fluid flow [6]. Furthermore, due to the spatial constraints, daughter cells either have to form as a chain, which is not necessarily the natural pattern for all bacteria, or they detach from the substrata and are lost in the flow [6]. Finally, the physical constraint in micro-channels may prevent significant aspects of cell division from occurring, such as snapping in rod-shaped actinomycetes [2].

Agar pad methods consist of bacterial cells sandwiched between a thin agar pad and a glass cover slip on top, which is then sealed. The advantages of this technique are that it is simple, relatively inexpensive to set up and there are no physical barriers allowing expansive growth. However, the environmental conditions cannot be manipulated which limits the variety of experiments that can be performed and the growth of aerobic cells may be affected in a sealed environment. The agar pad itself is also prone to desiccation over time hampering longer term experiments [6]. This technique is therefore best suited to short time studies where no manipulation of the culture conditions is required.

Time-lapse imaging of bacteria using existing agar pad methods have been used successfully to study a number of bacteria including *Escherichia coli *[7], *Corynebacterium glutamicum *[2]*, Vibrio cholerae *[1], *Caulobacter crescentus *[3] and *Pseudomonas aeruginosa *[5]. However, time-lapse imaging movies of the slower growing mycobacteria using existing agar pad methods are yet to be reported. Here we report the development of a modified agar pad method which overcomes some of the limitations of current protocols and present bacterial imaging and movie data obtained using this method.

## Materials and methods

*Mycobacterium smegmatis *mc^2^155, and *Mycobacterium bovis *BCG Pasteur were grown aerobically at 37°C with shaking at 180 rpm and 100 rpm, respectively, in Hartmans-de Bont minimal media [8]. The bacterial strain for live-cell imaging was grown to mid-log phase and a 200 μl aliquot of the bacterial suspension applied to an uncoated 35 mm petri dish that has a 15 mm diameter circle removed from the bottom of the dish and a borosilicate glass cover slip fixed to the underside of the dish creating a shallow 15 mm diameter well (commercially available from Matek). After 5 minutes, the liquid was removed by aspiration leaving a thin film of bacterial cells attached to the cover slip. A 3 ml aliquot of molten (37°C) top agar (standard growth media containing 0.6% Noble agar (Sigma)) was then added to the dish. The dish was left at room temperature for 45 mins to ensure complete solidification of the agar before mounting the specimen on the microscope within Perspex housing to maintain an ambient temperature of 37°C. The cells were viewed and images captured using a Zeiss Axiovert 200 inverted wide-field microscope fitted with an EM-CCD (C9100-02) camera (Hammamatsu). Automated image capture was performed using SimplePCI Compix software.

## Results and discussion

Our aim was to develop a simple and reliable method for single-cell time-lapse imaging of bacteria that could be used with both bright-field and fluorescence microscopy, permitted manipulation of the media during the experiment and allowed imaging of the slower growing mycobacteria.

**Figure 1:**
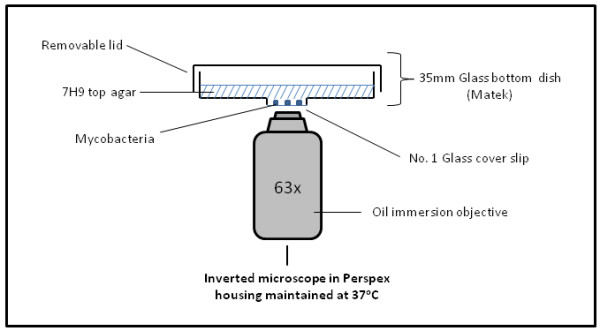
**Apparatus for live-cell imaging using the modified agar pad method**. Cells are seeded onto the glass cover slip of a 35 mm glass-bottomed dish. Agar (+/- dye, antibiotic, inducer) is then added to the dish and the cells viewed from below using an inverted microscope. Supplementation of the agar can also be achieved mid-experiment by removal of the lid and application of the supplement directly to the cell surface of the agar which can diffuse through the agar pad to the cells.

Similar to previous methods, in our procedure the cells are also sandwiched between a cover slip and an agar pad, but the agar pad is on top of the cover slip (rather than the reverse) and the cells viewed from below using an inverted microscope. A critical modification to existing methods is that the agar pad is made by adding a small volume of molten agar into a commercially available glass-bottomed dish (a standard 35 mm petri dish with a 15 mm diameter circle removed from the bottom and a borosilicate glass cover slip fixed to the underside of the dish creating a shallow 15 mm diameter well) (Figure 1). The molten agar concentration was optimised to give the lowest percentage agar that would remain solid when incubated at 37°C for imaging, maximising diffusion rates through the agar pad. The agar pad did not show signs of desiccation and was thin enough to facilitate sufficient oxygen exchange required for mycobacterial growth even over longer term experiments (>65 hours).

**Figure 2:**
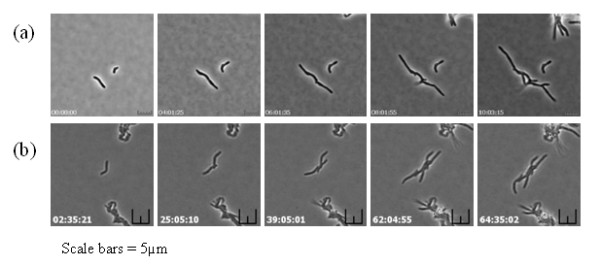
**Live cell images obtained by the modified agar pad method of (a) *M. smegmatis *and (b) *M. bovis *BCG**. (Scale bars = 5 μm). Time-lapse movies can be viewed in the additional files [Additional Files 1 &2].

These modifications to existing approaches provided optimal conditions for subsequent time-lapse imaging. However, during optimisation of the bright-field time lapse imaging procedure, the greatest challenge encountered was to ensure in-focus images were captured for each time point over the entire experiment, a problem caused by cell movement in the Z-plane and long experiment times due to the slow growth of mycobacteria. This challenge was overcome by using the autofocus function of the microscopy software to select a Z-plane and then capturing a stack of images around that plane. An in-focus image was manually selected at each time-point and converted into an .avi movie file using SimplePCI software. The method was applied to two different mycobacterial species and the images collected were combined to produce time-lapse images and movies of bacterial cell growth for *M. smegmatis *(Figure 2a & Additional File 1) and *M. bovis *BCG (Figure 2b & Additional File 2). The imaging and movie data obtained for mycobacteria showed some interesting and unique features compared to other rod-shaped bacteria, such as asymmetric cell growth and the lack of a uniform cell size that undergoes division. These issues are currently under further investigation.

**Figure 3:**
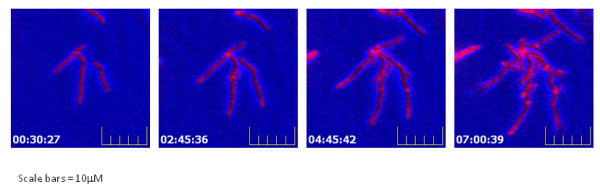
**Uptake by *M. smegmatis *of FM5-95 from 7H9 top agar during growth**. Cells were imaged at 37°C under 7H9 top agar supplemented with 2 μg/ml FM 5-95. Images were captured using a 560 nm excitation filter every 15 mins over 15 hrs with 50% of the maximum light intensity and the camera sensitivity set to 175. Time of image capture is shown (Scale bars = 10 μm).

This method potentially permits the addition of compounds, such as fluorescent dyes or antibiotics, to the media either at the start of the experiment by directly adding the substance to the molten agar or mid-experiment by application onto the agar pad surface and allowing diffusion through the agar to the cells. In order to confirm that compounds could be taken up from the agar during growth, the fluorescent membrane dye FM5-95 (2 μg/ml) was added to the agar at the start of the experiment and the cells seeded as described. Figure 3 shows that the cells have taken up the FM5-95 dye from the top agar during growth as the new daughter cells produced are all stained.

## Concluding remarks

We have developed a relatively simple and inexpensive method for time-lapse imaging single mycobacterial cells, which can also be applied to most other bacterial species. Fundamental issues such as bacterial growth patterns, protein localisation dynamics, antibiotic action and bacterial persistence could be studied using this technique. This method allows imaging over longer times (>65 hours), can be used in combination with fluorescence microscopy and permits the manipulation of environmental conditions during the imaging process. Furthermore, with the availability of multi-well glass-bottomed plates and automated microscope stages, this method is amenable to higher throughput analyses.

## Competing interests

The authors declare that they have no competing interests.

## Authors' contributions

GJ developed the technique and performed the research. KW and BR contributed to the design and trouble shooting of the procedure. KW wrote the manuscript. All authors read and approved the final manuscript.

## Supplementary Material

Additional file 1 **Time lapse movie of *M. smegmatis *growth using the modified agar pad method**. The optimised brightfield live cell imaging method was applied to study *M. smegmatis *growth. Cells were imaged every five minutes over 12 hrs and movies constructed from the time-lapse images using SimplePCI Compix software. Click here for file

Additional file 2 **Time lapse movie of *M. bovis *BCG growth using the modified agar pad method**. The optimised brightfield live cell imaging method was applied to study the growth of the slow growing mycobacteria, *M. bovis *BCG. Cells were imaged every hour over 68 hrs and movies constructed from the time-lapse images using SimplePCI Compix software. Click here for file
